# Are We Truly Preparing Students for Financial Success? Insights from Economic Education Curricula in Indonesia

**DOI:** 10.12688/f1000research.168866.1

**Published:** 2025-11-21

**Authors:** Dwi Nanda Akhmad Romadhon, Hari Mulyadi, Toni Heryana, Ismeirita

**Affiliations:** 1 Universitas Pendidikan Indonesia, Bandung, West Java, Indonesia

**Keywords:** Financial Literacy, Economics Education, Curriculum Development, Higher Education

## Abstract

**Background:**

Financial literacy is an essential competency for university students, especially those in economics education programs, as it supports effective money management and responsible decision-making. However, higher education curricula in Indonesia still emphasize theoretical aspects over practical financial skills, raising concerns about whether graduates are adequately prepared for financial challenges.

**Methods:**

This study employed a qualitative content analysis to evaluate the integration of financial literacy within economics education curricula in Indonesia. Data were collected through curriculum document reviews, semi-structured interviews with economics educators, and student surveys. The analysis focused on identifying the extent of financial literacy integration and perceptions of curriculum effectiveness.

**Results:**

The findings indicate that while fundamental topics such as budgeting and savings are covered, critical elements including investment diversification, risk management, and digital financial tools remain underdeveloped. Students expressed a strong demand for more experiential learning approaches, such as case studies, financial simulations, and collaboration with industry partners. The results highlight a gap between theoretical instruction and the practical competencies required for effective financial decision-making.

**Conclusions:**

The study concludes that strengthening economics education curricula requires the integration of applied financial literacy training, digital financial education, and industry collaboration to better prepare students for personal and professional financial challenges. While this research provides valuable insights, its qualitative scope presents limitations. Future studies with broader institutional coverage and quantitative approaches are recommended to generate more generalizable findings.

## Introduction

Financial literacy is a crucial competency for university students, particularly those enrolled in economics education programs. Financial literacy equips students with the knowledge to manage their finances effectively, including budgeting, saving, investing, and managing debt ( Zhou et al., 2024). Beyond enabling individuals to manage their personal finances effectively, financial literacy fosters informed decision-making in broader economic contexts. As financial markets become more complex and interconnected, the ability to understand financial principles and apply them in daily life is essential for long-term financial security and economic stability.

In Indonesia, financial literacy remains a widespread challenge, with a significant portion of the population lacking fundamental financial knowledge and decision-making skills. Many individuals struggle with essential aspects of financial management, including budgeting, saving, debt management, and investment planning. Limited financial awareness among students and graduates often leads to poor financial decisions, which can have long-term implications for their personal and professional lives. Consequently, higher education institutions play a pivotal role in equipping students with the necessary skills to navigate financial complexities effectively. Research indicates that financial knowledge and skills acquired through HEIs positively influence students’ financial attitudes and behaviors. For example, a study in Peru found that financial education significantly improved students’ financial attitudes, leading to more informed financial decisions ( Loza et al., 2024).

However, integrating financial literacy into economics education curricula presents several challenges. Existing curricula tend to emphasize theoretical knowledge over practical financial skills, leading to a disconnect between what students learn in academic settings and the financial realities they face. While students may develop a foundational understanding of economic concepts, they often lack exposure to real-world financial practices such as financial planning, risk assessment, and long-term investment strategies. Structured competitions where students analyze current economic issues using economic tools can foster creativity, collaboration, and critical thinking, while also reinforcing foundational concepts ( Beaudin et al., 2017). This misalignment raises concerns about whether higher education adequately prepares students for financial independence and economic participation.

Addressing these gaps requires a comprehensive review of existing curricula to assess their effectiveness in fostering financial literacy. Enhancing financial education at the university level should go beyond introducing standalone courses; it necessitates the integration of experiential learning methods, case studies, and applied financial decision-making exercises. By embedding these elements into the curriculum, higher education institutions can better equip students with the competencies required to make sound financial decisions, both personally and professionally. Integrating critical thinking on sustainable finance into finance courses can help students incorporate sustainable investment decisions and promote the United Nations’ Sustainable Development Goals (SDGs) ( Yu & Zhang, 2025).

Despite the increasing recognition of financial literacy as a critical skill for university students, a significant gap exists between the competencies required for financial independence and the content delivered in economics education curricula. Many students enter higher education with limited financial knowledge, yet they are expected to make complex financial decisions related to tuition fees, student loans, savings, investments, and long-term financial planning. Theoretical knowledge alone is insufficient to prepare students for these challenges, as financial literacy requires both conceptual understanding and practical application.

A key concern is whether business and economics education curricula in Indonesian universities are structured to adequately develop students’ financial literacy. There is no specific financial literacy curriculum in Indonesian universities, which suggests a need for a more structured approach ( Rafinda, 2022; Sari, 2024). Many programs focus on macroeconomic and microeconomic theories while placing limited emphasis on practical financial skills, such as managing personal finances, understanding credit and debt, analyzing investment opportunities, and mitigating financial risks. This gap raises important questions about the relevance and effectiveness of current curricula in addressing real-world financial challenges faced by graduates.

Moreover, the disconnect between academic instruction and real-world financial demands suggests that traditional lecture-based approaches may not be the most effective way to teach financial literacy. Without exposure to experiential learning, such as financial simulations, case-based teaching, and applied economic decision-making, students may struggle to translate theoretical concepts into actionable financial strategies. This gap in pedagogical approaches further reinforces concerns about whether economics education at the university level adequately prepares students to navigate increasingly complex financial landscapes.

Given these challenges, this study seeks to critically examine the extent to which business education curricula in Indonesian universities support the development of financial literacy among students. Specifically, it aims to identify gaps in curriculum content, pedagogical approaches, and skill development opportunities to determine whether current educational practices align with students’ financial literacy needs in today’s dynamic economic environment. By addressing these gaps, higher education institutions can play a more proactive role in ensuring that students graduate with the financial competencies necessary for both personal financial well-being and broader economic participation.

This study aims to critically examine the extent to which business education curricula in Indonesian universities support the development of financial literacy among students in economics education programs. Given the increasing complexity of financial decision-making in both personal and professional contexts, it is essential to assess whether current curricula equip students with the necessary knowledge and practical skills to navigate financial challenges effectively.

Specifically, this research will analyze curriculum content, pedagogical approaches, and the integration of financial literacy components within economics education programs. The study will evaluate the extent to which financial literacy topics such as budgeting, financial planning, credit management, investment literacy, risk assessment, and consumer financial decision-making are embedded within course structures. This assessment will be conducted with reference to national and international financial literacy standards, ensuring alignment with best practices in financial education. The National Standards for Financial Literacy outline the essential knowledge, understanding, and skills students need to learn about personal finance. These standards guide educators in developing curricula and educational materials ( Bosshardt & Walstad, 2014).

Furthermore, the study seeks to identify existing gaps between theoretical instruction and practical financial competency development. By mapping curriculum coverage against students’ financial literacy needs, the research will highlight areas where improvements are required, whether through curriculum revision, instructional innovation, or experiential learning opportunities, such as simulations, case studies, and applied financial decision-making exercises.

Ultimately, this study aims to provide evidence-based recommendations for higher education policymakers, curriculum designers, and educators to refine and enhance financial literacy education within economics programs. By bridging the gap between financial literacy instruction and real-world financial challenges, this research aspires to contribute to the development of a more financially literate generation of graduates capable of making informed and responsible economic decisions in an increasingly dynamic financial environment.

This research holds both academic and practical significance. Academically, it provides insights into how the current business education curricula in Indonesia support financial literacy development. It contributes to the existing body of knowledge on curriculum effectiveness and financial literacy education, offering a foundation for further research on innovative pedagogical approaches.

Practically, the findings are expected to assist education policymakers, curriculum developers, and university administrators in designing curricula that are more responsive to students’ financial literacy needs. By incorporating more practical financial literacy components, universities can better prepare students to make informed financial decisions that impact their personal financial stability and professional success.

By addressing these educational gaps, this study advocates for a more comprehensive and application-oriented approach to financial literacy education within economics programs. The insights generated will serve as a guide for universities to integrate real-world financial literacy training into their curricula, ultimately enhancing students’ ability to navigate financial challenges effectively.

## Literature review

### Financial literacy standards

Financial literacy education is guided by well-established standards to ensure individuals acquire essential financial competencies. In Indonesia, the National Financial Literacy Standards, developed by the Financial Services Authority (Otoritas Jasa Keuangan/OJK), serve as a fundamental framework for financial education. These standards cover key areas such as personal financial management, savings, investment, risk management, and financial protection, aiming to equip individuals with the knowledge and skills necessary to make informed financial decisions. The standards also integrate financial literacy with numeracy skills, recognizing the importance of mathematical abilities in understanding and managing financial matters. This integration is particularly relevant in educational settings, where financial literacy is embedded in subjects like mathematics and economics ( Sari, 2024). A curriculum aligned with these standards is expected to produce graduates who are financially literate and capable of navigating personal and professional financial challenges effectively.

Comparatively, other countries have taken more structured and innovative approaches to financial literacy education. China has shown significant advancements in financial literacy, particularly among teenagers, due to innovative educational approaches. This has positioned China favorably in financial literacy rankings among G-20 countries ( Klepikova, 2018). This approach provides students with hands-on experiences that bridge the gap between theoretical knowledge and practical financial decision-making. Indonesia could benefit from adopting similar models, ensuring that financial literacy education goes beyond theoretical discussions and actively prepares students for real-world financial responsibilities.

### Previous studies on financial literacy

Extensive research has demonstrated the critical role of financial literacy in shaping students’ financial behavior and decision-making. Higher financial literacy is strongly related to sound financial planning and decision-making. Individuals with better financial knowledge, behavior, and attitudes are more likely to engage in effective financial planning and make informed financial decisions ( Singh & Thakur, 2024). Their study emphasizes the importance of early exposure to financial education, arguing that financial literacy should be embedded within formal education systems to cultivate responsible financial habits from a young age.

Furthermore, research has shown that traditional, lecture-based financial education often fails to engage students effectively. Interactive teaching methods, such as financial simulations, gamification, and multimedia learning tools, have been found to enhance students’ financial understanding and retention. Experiential learning has been linked to positive changes in financial behavior. For instance, a study in rural Uganda found that active learning methods led to significant improvements in savings and investment outcomes compared to traditional lecturing ( Kaiser & Menkhoff, 2022).

### Economic literacy and financial behavior

Economic literacy serves as a fundamental pillar that supports financial literacy. conomic literacy contributes to wealth accumulation by helping individuals understand and navigate economic variables that affect their financial well-being. Research indicates that knowledge in areas such as risk management and debt literacy, which are rooted in economic principles, significantly impacts wealth accumulation ( Sekita et al., 2022). A strong grasp of economic principles enables students to understand broader financial concepts, including inflation, interest rates, credit systems, and economic cycles, which are crucial for making informed financial decisions. Studies suggest that students with higher levels of economic literacy are more likely to engage in responsible financial behaviors, such as budgeting, saving, and prudent debt management ( Haryono et al., 2022; Jali et al., 2023; Kustiandi et al., 2024).

The impact of integrating economic literacy into university curricula and found a positive correlation between economic understanding and financial attitudes ( Mandell & Klein, 2009). Their findings suggest that students who receive structured education in economic literacy are more likely to exhibit financial behaviors that contribute to long-term financial security, such as prioritizing savings and avoiding excessive debt. This indicates that enhancing economic literacy within higher education curricula can significantly improve students’ financial well-being and decision-making capabilities.

### Research gap

Despite the extensive body of research on financial literacy, there remains a critical gap in studies analyzing financial literacy curricula within economics education programs at Indonesian universities. Most existing research focuses on general financial literacy levels, behavioral aspects of financial decision-making, or interventions aimed at improving financial knowledge. However, limited attention has been given to evaluating the structure, content, and effectiveness of business education curricula in fostering financial literacy among university students.

This study seeks to address this gap by assessing the extent to which economics education curricula in Indonesian universities integrate financial literacy components. It aims to evaluate whether existing curricula sufficiently equip students with practical financial skills or if they primarily focus on theoretical economic concepts. By examining curriculum alignment with national and international financial literacy standards, this research provides valuable insights for policymakers, educators, and curriculum developers in enhancing financial literacy education at the university level.

## Methodology

### Research design

This study adopts a qualitative research approach with content analysis as the primary method to evaluate the extent to which financial literacy is embedded within business education curricula in Indonesian universities. The research aims to assess the alignment between curriculum content and the National Financial Literacy Standards, identifying gaps and areas for improvement. A qualitative approach allows for an in-depth exploration of curriculum structure, instructional materials, and educators’ perspectives on financial literacy education.

### Data collection

Data were collected through document analysis, focusing on various instructional materials, including syllabi, semester learning plans (RPS), textbooks, and other teaching resources used in economics education programs across selected Indonesian universities, Universitas Jambi and Universitas Pendidikan Indonesia. These documents were examined to determine the coverage of financial literacy concepts and the extent to which they emphasize practical financial skills, such as financial planning, debt management, and investment literacy.

In addition to document analysis, semi-structured interviews were conducted with lecturers responsible for teaching economics and financial literacy-related courses. These interviews aimed to capture educators’ perspectives on the integration of financial literacy in their curricula, including challenges, teaching methodologies, and potential areas for enhancement.

Furthermore, a short student survey was administered to gather learners’ perceptions of financial literacy coverage and the usefulness of applied activities, such as case studies, simulations, and financial technology (fintech) tools.

The combination of document analysis, interviews, and student surveys provides a comprehensive understanding of both the intended curriculum (as reflected in course materials) and the enacted curriculum (as interpreted and delivered by lecturers).

### Ethical considerations and informed consent

This study involved interviews with lecturers and reviews of curriculum documents; no sensitive student records were used. All participants were adults (≥18 years), and no minors were included. Verbal informed consent was obtained from all participants prior to interviews (and student surveys, where applicable). Written consent was not feasible because several participants were unfamiliar or uncomfortable with signing formal research documents in this context, which could have created unnecessary barriers to participation. To ensure ethical compliance, verbal consent was audio-recorded at the start of each session. Participants were informed about the study objectives, voluntary participation, the right to withdraw at any time without consequences, and confidentiality safeguards.

All datasets were de-identified before analysis and deposition in accordance with the HIPAA Safe Harbor method; direct identifiers (e.g., personal names, institutional roles) were removed.

### Data analysis framework

Data analysis was conducted using MAXQDA, a qualitative data analysis software that facilitated systematic coding and thematic categorization of curriculum content. The software offers methodical methodologies for the extraction of codes and themes from qualitative data, a process that is crucial for conducting thematic analysis ( Alonso et al., 2021). The analysis framework was based on the revised Bloom’s Taxonomy, which classifies learning objectives into cognitive levels, ranging from basic knowledge acquisition to higher-order thinking skills such as application and analysis. This taxonomy was used to evaluate whether the curriculum fosters merely theoretical understanding or also develops students’ practical financial decision-making skills.

The coded data from document analysis and interviews were then compared against the National Financial Literacy Standards to identify discrepancies and gaps between the intended curriculum and the competencies expected for financial literacy. Key themes emerging from the qualitative analysis included the depth of financial literacy integration, the emphasis on theoretical versus practical content, and the challenges faced in implementing financial literacy education within economics education programs.

### Validity and reliability

To ensure validity and reliability, this study employed data triangulation, wherein findings from document analysis, interviews, and financial literacy standards were cross-validated. Triangulation serves to substantiate research conclusions by verifying that diverse methodologies or observers examining the identical phenomenon yield congruent outcomes. (A. Nightingale, 2009; A. J. Nightingale, 2019). Comparing data from multiple sources enhanced the credibility of the study by ensuring that conclusions were not based on a single type of evidence.

Additionally, expert discussions were conducted with financial literacy and education specialists to validate the coding framework and interpretations of the data. This process helped reduce researcher bias and ensure that the analysis accurately reflects the actual state of financial literacy education in Indonesian universities. Maintaining consistency in the coding and thematic analysis further reinforced the study’s reliability, ensuring that findings are replicable and aligned with qualitative research rigor.

## Results and discussion

### Content analysis findings

The analysis of business education curricula in Indonesian universities revealed that while financial literacy topics are integrated into the coursework, their depth and comprehensiveness vary significantly. Certain core topics, such as financial planning and savings, are frequently covered, whereas other essential components like investment diversification and risk management receive limited attention. This imbalance suggests that while students may develop a basic understanding of financial management, they may not be adequately prepared for complex financial decision-making in real life scenarios.

The findings are summarized in Table 1, which illustrates the frequency and perceived importance of financial literacy topics within the curriculum.

**Table 1.** Coverage of financial literacy topics in business/economics education curricula.

Financial literacy topic	Frequency in curriculum	Importance for financial literacy
Financial Planning	High	Very Important
Expense Management	Medium	Important
Savings and Investment	High	Very Important
Debt Management	Medium	Important
Investment Diversification	Low	Very Important
Risk Management	Low	Very Important

From the table, it is evident that while financial planning and savings are prioritized, investment diversification and risk management are underrepresented, despite their critical role in financial decision-making. This misalignment could result in graduates who lack the necessary competencies to manage financial risks effectively, particularly in a rapidly evolving financial landscape.

**Figure 1.**
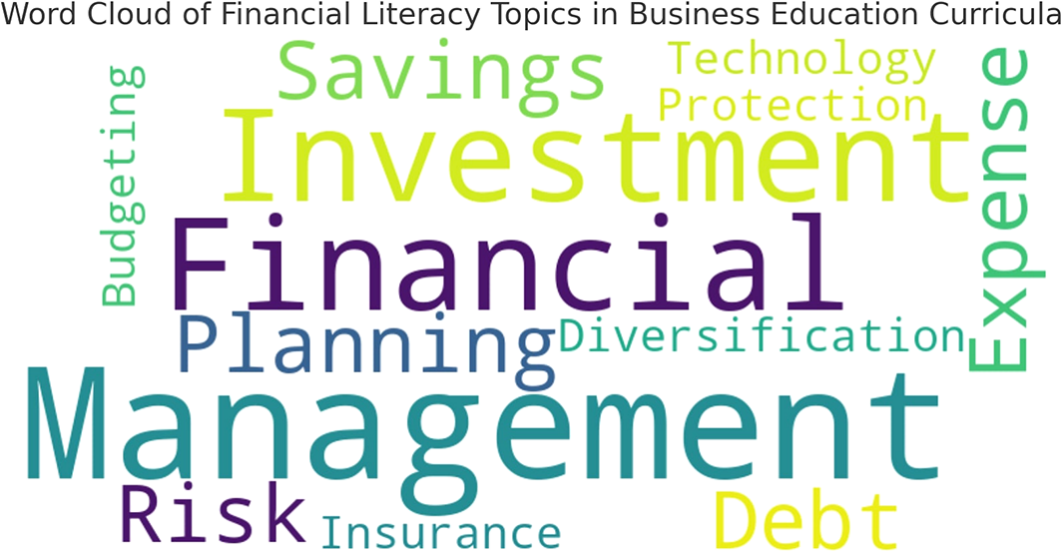
Word cloud of financial literacy topics in business education curricula.

To further illustrate the focus areas in financial literacy education, Figure 1 presents a word cloud generated from curriculum analysis, highlighting the frequency of financial literacy related terms in course materials.

The word cloud reflects the emphasis placed on various financial literacy topics within the curriculum. Larger words indicate topics that appear more frequently in teaching materials, while smaller words represent less commonly covered areas.

This visualization reinforces the findings from Table 1, demonstrating that fundamental topics like financial planning and savings dominate the curriculum, whereas more advanced areas like investment diversification and financial risk management receive comparatively little attention.

### Comparison with national financial literacy standards

A comparison between the curriculum content and the National Financial Literacy Standards reveals several gaps in financial literacy education. The National Standards for Financial Literacy delineate critical knowledge and competencies requisite for personal financial management, with the objective of assisting educators in the formulation of curricular resources. These standards underscore the significance of economic principles and decision-making capabilities as fundamental components of financial literacy ( Bosshardt & Walstad, 2014). While budgeting, savings, and debt management are well-represented, topics such as insurance protection, investment diversification, and financial risk assessment are either insufficiently covered or entirely absent in many programs. This gap suggests that students may graduate without a comprehensive understanding of financial risk mitigation strategies, potentially leaving them vulnerable to poor financial decision-making.

**Figure 2.**
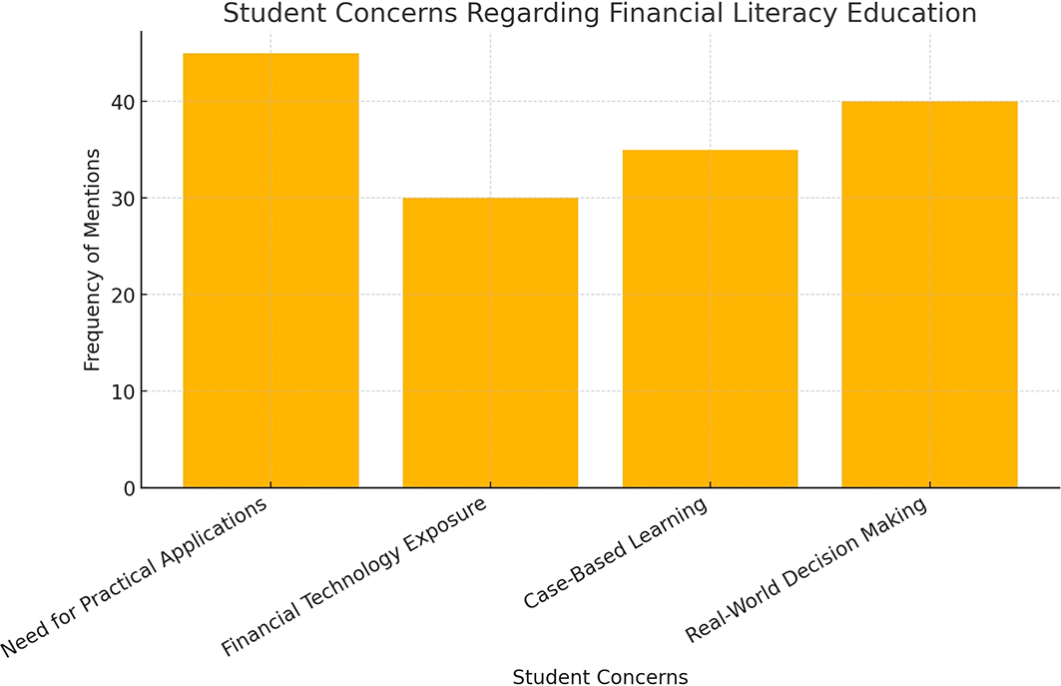
Comparison of curriculum coverage with national financial literacy standards.

The extent of these gaps is further illustrated in Figure 2, which compares the coverage of financial literacy topics in business education curricula against the expectations set by national standards.

This bar chart illustrates the discrepancies between financial literacy topics covered in business curricula and the national standards. The lower coverage in areas such as investment diversification and risk management indicates potential areas for curriculum improvement.

The findings suggest that while basic financial literacy competencies are covered, the lack of advanced financial concepts and digital financial literacy training poses challenges for students navigating real-world financial decision-making.

### Student perspectives on financial literacy education

Interviews with students provided additional insights into their experiences with financial literacy education. While most students acknowledged that the curriculum helped them understand core financial concepts, many expressed concerns about the lack of practical applications. They emphasized the need for more hands on learning experiences, such as case studies, financial simulations, and the use of digital financial tools.

**Figure 3.**
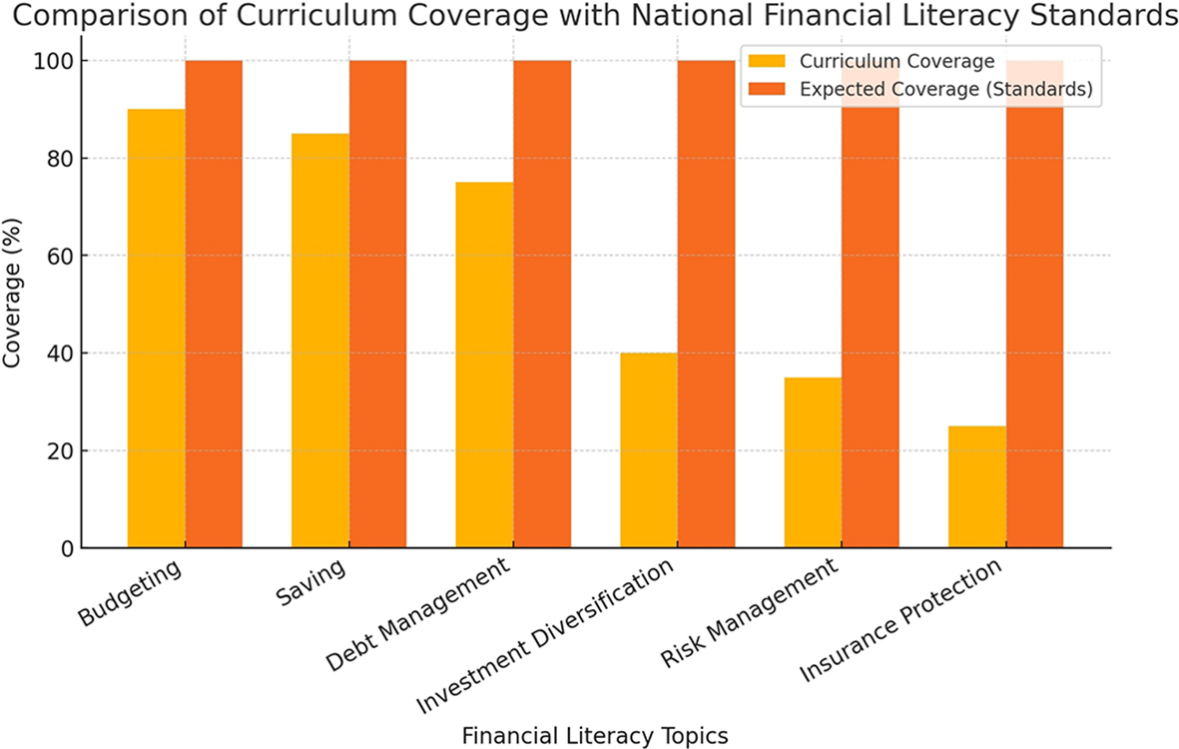
Student concerns regarding financial literacy education.

The key concerns raised by students are summarized in Figure 3, which presents a coding frequency analysis of student interviews conducted in MAXQDA.

This chart highlights the most frequently mentioned concerns from student interviews. The most common issues include the need for practical applications, financial technology exposure, and case-based learning approaches.

The majority of students expressed interest in learning about financial technologies, particularly investment applications, mobile banking platforms, and financial planning software. Many felt that these tools were essential for real-world financial decision making but were not sufficiently covered in their courses.

## Discussion

### Implications for curriculum design

The findings of this study indicate that while financial literacy is integrated into business education curricula, significant gaps persist, particularly in investment strategies, risk management, and financial technology applications. For instance, financial literacy workshops and service-learning projects have been implemented in finance courses to provide real-world applications and improve financial literacy skills among students ( Buchanan, 2014; Rosacker & Rosacker, 2016). These deficiencies suggest that current curricula may not fully equip students with the necessary knowledge and skills for financial independence and professional financial decision-making. To address these shortcomings, curricula should be expanded to cover a broader range of financial literacy topics that align with real world financial challenges.

Beyond expanding content coverage, there is a need to transform the approach to financial literacy education. The current reliance on theoretical instruction limits students’ ability to apply financial concepts in practical settings. A shift towards experiential learning methods, such as financial case studies, real world simulations, and industry collaborations, would enhance students’ financial competencies. In particular, Financial Trading Rooms (FTRs) and trading simulators provide students with real-time market information, allowing them to apply classroom theories to practical scenarios. This hands-on experience has been shown to improve students’ understanding of financial concepts and increase their engagement and satisfaction with the learning process ( Bakoush, 2022; Noreen, 2022; Sharma et al., 2018). By integrating these approaches, students can engage in financial decision-making scenarios that mirror real life financial complexities, better preparing them for financial challenges beyond the academic environment.

### Recommendations for enhancing financial literacy education

To strengthen financial literacy education in business curricula, several improvements must be considered. First, universities should integrate essential but underrepresented topics, such as investment diversification, risk management, and insurance protection, into their financial literacy programs. These topics are critical for ensuring that students develop a comprehensive understanding of financial decision-making and long-term financial security. The acquisition of financial literacy equips students with the necessary tools to evade fraudulent schemes and to engage in discerning decision-making concerning insurance and various financial instruments ( Bhattacharya & Sarkar, 2023).

Additionally, financial literacy education should be made more interactive and engaging. Traditional lecture based instruction should be complemented with financial simulation games, investment management exercises, and financial planning software. Such interactive methods would allow students to develop hands on experience in financial decision making, reinforcing their understanding of financial concepts. The implementation of digital instruments and virtual financial simulations can markedly augment financial literacy among students. Caregivers and instructors favor these approaches as they render the educational experience more stimulating and efficacious ( Murugiah et al., 2023).

Modern financial literacy also requires competency in digital financial tools. Given the increasing role of financial technology in personal and professional finance, curricula should include training on investment applications, online banking platforms, and budgeting software. This would ensure that students acquire the necessary digital skills to manage financial transactions effectively in an increasingly digital economy.

Collaboration with industry professionals is another critical aspect of improving financial literacy education. Integrating practical teaching content with actual corporate projects and job requirements significantly enhances students’ financial practical abilities and competitiveness in the job market. This approach has been successfully implemented through school-enterprise cooperation, which develops rich teaching resources and establishes competency-focused assessment systems ( Li, 2023). Partnerships with financial professionals, investment advisors, and banking practitioners can provide students with insights into real-world financial decision making. Industry collaborations through guest lectures, workshops, and mentorship programs would enable students to connect theoretical learning with industry practices, fostering a deeper understanding of financial management strategies.

Universities should also encourage experiential learning by developing case based learning modules, collaborative projects, and hands on financial planning workshops. Workshops that emphasize useful skills, like budgeting, may be quite successful in getting students ready for certain job requirements ( Morrissey et al., 2017; Okoli et al., 2019). These initiatives would expose students to real financial scenarios, allowing them to practice financial decision-making in a structured environment before encountering these challenges in professional settings.

By implementing these recommendations, financial literacy education in business curricula can evolve into a more holistic and practical learning experience that ensures students develop both theoretical knowledge and practical financial skills.

### Impact on students’ financial behavior

A well designed financial literacy curriculum has the potential to significantly influence students’ financial behavior by equipping them with the skills and knowledge necessary to navigate financial challenges effectively. Several studies indicate that financial literacy programs can lead to positive changes in financial behavior. For example, a financial literacy course for low-income individuals resulted in increased financial knowledge and positive financial behaviors, such as better money management and reduced negative behaviors like overdrawing accounts ( Reich & Berman, 2015). With a more comprehensive financial education, students are expected to develop better personal financial habits, including responsible budgeting, strategic saving, and informed investing. These practices contribute to greater financial security, reducing the likelihood of financial instability in the future.

Beyond personal financial management, improved financial literacy education can enhance students’ ability to make sound financial decisions, particularly in areas such as credit management, debt repayment strategies, and investment planning. With a deeper understanding of these concepts, students can avoid common financial pitfalls and adopt financial strategies that promote long term financial stability. Higher financial literacy levels are associated with better financial practices, such as increased savings and responsible debt management, which enhance economic resilience at the household level ( Katnic et al., 2024).

The integration of digital financial tools into curricula is expected to increase students’ ability to utilize financial technology for better financial planning and wealth management. Exposure to fintech applications, automated budgeting tools, and investment platforms will ensure that students are well-equipped to manage their finances using modern digital resources. This digital literacy will be crucial in an era where financial transactions and investment opportunities are increasingly technology driven. Digital financial literacy significantly influences financial behavior, including the use of digital financial services such as mobile payments, online borrowing, and online financial products. This impact is more pronounced with the complexity of digital finance ( Yang et al., 2023).

Another expected outcome of enhanced financial literacy education is the development of greater financial resilience and risk awareness. By incorporating topics such as risk management and insurance protection, students will be better prepared to assess financial risks and make informed decisions to safeguard their financial well being. Understanding risk diversification and financial planning strategies will help students make smarter investment choices and manage financial uncertainties more effectively.

Ultimately, strengthening financial literacy education in business curricula will not only benefit individual students but also contribute to broader financial stability within society. Graduates who possess strong financial knowledge and decision making skills will be better positioned to navigate financial challenges and contribute to economic resilience. A well structured curriculum that integrates theoretical knowledge with practical financial competencies will ensure that students become financially responsible and economically empowered individuals in an increasingly complex financial landscape. By fostering a financially literate generation, universities can play a vital role in promoting responsible financial behavior and ensuring long-term economic stability.

## Conclusion

### Summary of findings

This study highlights that business education curricula in Indonesian universities incorporate several critical aspects of financial literacy, particularly in areas such as financial planning and savings. However, the analysis also reveals notable gaps, particularly in investment diversification and risk management, which are essential for equipping students with comprehensive financial decision making skills. The findings further indicate that students perceive existing financial literacy education as overly theoretical, with a strong demand for more practical, interactive learning experiences such as simulations, case studies, and exposure to financial technologies. Effective financial literacy education should involve practical work, such as using board games, digital applications, and real-life scenarios to make learning more engaging and applicable ( Alamin et al., 2022; Sconti, 2022; Sudakova, 2018). Addressing these gaps in curricula is crucial to ensuring that graduates are fully prepared to navigate complex financial landscapes in both their personal and professional lives.

### Future research directions

The findings of this study open several avenues for further research. One potential area of exploration is the long-term impact of financial literacy education on students’ financial behavior after graduation. Investigating whether students who receive more comprehensive financial literacy training exhibit better financial habits, stronger financial resilience, and higher financial well-being over time would provide valuable insights into the effectiveness of financial education initiatives. Additionally, future research could focus on assessing the effectiveness of interactive teaching methods, such as financial simulations, gamification, and technology based learning, in improving financial literacy outcomes. Comparative studies between traditional lecture-based approaches and experiential learning strategies would help identify best practices for financial literacy instruction in higher education. Traditional methods involve structured courses that cover a range of financial topics. For example, a semester-long course on family economics showed significant learning gains in financial literacy ( Maurer & Lee, 2011).

### Practical implications

The findings of this study provide a strong foundation for revising business education curricula to better align with students’ financial literacy needs. By integrating a broader range of financial literacy topics and adopting more experiential learning approaches, universities can enhance students’ financial competencies and preparedness for real world financial challenges. Strengthening financial literacy education will not only benefit students personally by improving their ability to manage personal finances and investments but will also contribute to their professional development, equipping them with skills that are increasingly relevant in modern economic environments.

By implementing curriculum improvements based on these findings, universities can take a more proactive role in fostering a financially literate and economically responsible generation. This, in turn, will have broader societal benefits, as graduates who possess strong financial literacy skills are more likely to make informed financial decisions, contribute to economic stability, and reduce financial vulnerabilities. Thus, revising and enhancing financial literacy education is not just an academic priority but also an essential step in ensuring long-term financial resilience at both individual and national levels.

## Ethics statement

The study complied with the principles of the Declaration of Helsinki. Given the minimal-risk, education-research context and the absence of sensitive personal data, formal institutional ethics approval was not required at the time of data collection under local regulations. All participants were adults (≥18 years) and provided verbal informed consent, which was audio-recorded. Verbal informed consent was obtained from all participants prior to interviews (and student surveys, where applicable). Written consent was not feasible because several participants were unfamiliar or uncomfortable with signing formal research documents in this context, which could have created unnecessary barriers to participation. To ensure ethical compliance, verbal consent was audio-recorded at the start of each session. Participants were informed about the study objectives, voluntary participation, the right to withdraw at any time without consequences, and confidentiality safeguards.

## Data Availability

The de-identified data supporting this study are available in the Zenodo repository at: https://doi.org/10.5281/zenodo.17182353 ( Romadhon, 2025). All datasets have been de-identified in accordance with the HIPAA Safe Harbor method; direct identifiers (e.g., names, institutional roles) were removed prior to analysis and deposition. The previously posted version containing identifiable fields has been withdrawn at our request, and only the fully de-identified dataset is now available. Data are available under the terms of the Creative Commons Attribution 4.0 International license (CC-BY 4.0).
