# Supplementary material for: Are We Truly Preparing Students for Financial Success? Insights from Economic Education Curricula in Indonesia
Source: F1000Res. 2026 May 29;14:1291. Originally published 2025 Nov 21. [Version 3] doi: 10.12688/f1000research.168866.3 (PMC13218411; doi:10.12688/f1000research.168866.3)
Supplement: Peer review history

# Reviewer report: Reviewer response for version 2

**DOI:** 10.5256/f1000research.197628.r475788

**Published:** 2026-05-18

**Authors:** Ratih Kusumawardhani

**Affiliations:** 1 Universitas Sarjanawiyata Tamansiswa, Yogyakarta, Special Region of Yogyakarta, Indonesia

The manuscript addresses a highly relevant and timely topic and demonstrates strong potential for contribution to the field of financial literacy and curriculum studies. However, to reach the standard expected for indexing in a high-quality international journal, the manuscript requires substantial improvement in theoretical grounding, methodological transparency, and analytical depth.

- Theoretical Framework
  - The study should be anchored in recognized frameworks (e.g., OECD financial literacy framework, experiential learning theory, competency-based education).
- Methodological Transparency
  - The coding process, validation, and sampling strategy require clearer and more detailed explanation.
- Analytical Depth in Discussion
  - The discussion section tends to restate results rather than interpret them.
  - The manuscript should explain *why* the identified gaps exist and their broader implications.
  - The authors should include real-world examples, case studies, or best practices.

Is the work clearly and accurately presented and does it cite the current literature?

Partly

If applicable, is the statistical analysis and its interpretation appropriate?

Partly

Are all the source data underlying the results available to ensure full reproducibility?

Yes

Is the study design appropriate and is the work technically sound?

Yes

Are the conclusions drawn adequately supported by the results?

Yes

Are sufficient details of methods and analysis provided to allow replication by others?

Partly

Reviewer Expertise:

My research expertise lies in Financial Management, with a particular emphasis on Financial Literacy and Digital Financial Literacy. My work explores the role of financial knowledge and digital capabilities in shaping financial behavior, financial inclusion, financial resilience, and performance outcomes, especially within the context of SMEs and households in emerging economies.

I confirm that I have read this submission and believe that I have an appropriate level of expertise to confirm that it is of an acceptable scientific standard, however I have significant reservations, as outlined above.

# Reviewer report: Reviewer response for version 2

**DOI:** 10.5256/f1000research.197628.r475790

**Published:** 2026-05-12

**Authors:** Mohammad Rakibul Islam Bhuiyan, Lisa Mani

**Affiliations:** 1 Begum Rokeya University, Rangpur City, Bangladesh; 2 Finance and Banking, Shahjalal University of Science and Technology School of Management and Business Administration (Ringgold ID: 309531), Sylhet, Sylhet, Bangladesh

Authors have addressed the comments successfully and meet the standard for the indexing smoothly. The citations or justification of the introduction and literature review must be more than 20 papers from the 2020-2026. There are a scare of citations and most the text is self writing which need to address it.

Is the work clearly and accurately presented and does it cite the current literature?

Yes

If applicable, is the statistical analysis and its interpretation appropriate?

Yes

Are all the source data underlying the results available to ensure full reproducibility?

Yes

Is the study design appropriate and is the work technically sound?

Yes

Are the conclusions drawn adequately supported by the results?

Yes

Are sufficient details of methods and analysis provided to allow replication by others?

Yes

Reviewer Expertise:

Digital Transformation, Machine Learning,

We confirm that we have read this submission and believe that we have an appropriate level of expertise to confirm that it is of an acceptable scientific standard.

## References

[1] : Are We Truly Preparing Students for Financial Success? Insights from Economic Education Curricula in Indonesia. *F1000Research* .2026;14: 10.12688/f1000research.168866.2 10.12688/f1000research.168866.2 PMC1321841142222133

# Reviewer report: Reviewer response for version 2

**DOI:** 10.5256/f1000research.197628.r475787

**Published:** 2026-05-05

**Authors:** Nurul Hidayana Mohd Noor

**Affiliations:** 1 Universiti Teknologi MARA, Seremban, Negeri Sembilan, Malaysia

1. The authors are advised to further elaborate on financial struggles and socio-economic issues among Indonesians by incorporating recent empirical evidence and statistical data. These statistics and empirical findings demonstrate that financial struggles in Indonesia are both systemic and significant, warranting deeper discussion and critical analysis in the manuscript.
2. The authors are encouraged to strengthen the discussion on the importance of financial literacy among students by incorporating more supporting literature. At present, the number of cited studies appears insufficient to convincingly establish the significance and urgency of this issue.
3. The authors are advised to clarify what is meant by the term “financial literacy standards.” It is currently unclear why the term standards is used instead of more commonly applied terms such as frameworks, competencies, or guidelines. The manuscript would benefit from a precise definition of this concept, including whether it refers to nationally or internationally recognized benchmarks, educational frameworks, or specific measurable competencies. Clarifying this will improve conceptual clarity and avoid potential ambiguity for readers.
4. The explanation of financial literacy in the manuscript is currently not sufficiently clear or well-developed. The authors are encouraged to strengthen this section by grounding their discussion in established models or existing theoretical frameworks. For instance, incorporating perspectives from recognized frameworks (e.g., OECD financial literacy framework) or relevant behavioural and economic theories would provide a more robust conceptual foundation. Clarifying the underlying dimensions, components, and theoretical basis of financial literacy will enhance the overall rigor and coherence of the study.
5. The authors state that the study adopts a qualitative approach; however, the methodology described appears to rely on a short survey instrument, which is typically associated with quantitative research. This creates a methodological inconsistency. The authors are advised to clarify the research design and justify the chosen approach. If the study is intended to be qualitative, more appropriate methods (e.g., interviews, focus groups, or open-ended responses with in-depth analysis) should be employed. Alternatively, if a survey was used, the study may be more accurately described as quantitative or mixed methods. Clear alignment between the research design, data collection methods, and analysis is necessary to ensure methodological rigor.
6. The section on Recommendations for enhancing financial literacy education requires further elaboration. At present, the discussion remains quite general and would benefit from more detailed explanation. The authors are encouraged to include concrete real-life examples, case studies, or best practices from existing programs or institutions to better illustrate how these recommendations can be implemented in practice. Incorporating such evidence will strengthen the applicability and practical relevance of the study.

Is the work clearly and accurately presented and does it cite the current literature?

Partly

If applicable, is the statistical analysis and its interpretation appropriate?

Yes

Are all the source data underlying the results available to ensure full reproducibility?

Yes

Is the study design appropriate and is the work technically sound?

Yes

Are the conclusions drawn adequately supported by the results?

Yes

Are sufficient details of methods and analysis provided to allow replication by others?

Yes

Reviewer Expertise:

youth studies, women studies, entreprenuership, management, environmental studies

I confirm that I have read this submission and believe that I have an appropriate level of expertise to confirm that it is of an acceptable scientific standard, however I have significant reservations, as outlined above.

# Response

**Published:** 2026-05-06

**Authors:** Dwi Romadhon

**Affiliations:** FPEB, Universitas Pendidikan Indonesia, Bandung, West Java, Indonesia

We sincerely thank the reviewer for the constructive and insightful comments. We have carefully revised the manuscript to address all concerns. Below we provide a detailed, point-by-point response.

**Comment 1: Insufficient empirical evidence on financial struggles in Indonesia**

**Reviewer concern:**

 Need stronger empirical/statistical grounding showing financial struggles are systemic.

**Response**

 We agree. The Introduction has been substantially strengthened by incorporating recent national-level empirical evidence.

**Revisions made**

 We added:

- **OJK National Financial Literacy Survey (SNLIK 2022/2024)**
  - Literacy vs inclusion gap (e.g., ~49% literacy vs >80% access)
- Evidence on:
  - Low investment participation
  - High consumer debt vulnerability
  - Youth financial fragility

**Inserted paragraph (example)**

 Recent national data indicate that financial literacy in Indonesia remains structurally limited. The OJK National Survey (SNLIK) reports that although financial inclusion exceeds 80%, financial literacy remains below 50%, indicating a significant capability–access gap. This mismatch suggests that many individuals participate in financial systems without adequate understanding, increasing vulnerability to poor financial decisions, over-indebtedness, and exposure to financial fraud.

**Impact**

- Strengthens **problem urgency**
- Moves from *normative claim → evidence-based claim*
- Improves **editorial credibility (Q1 expectation)**

**Comment 2: Insufficient literature on importance of financial literacy**

**Reviewer concern:**

 Literature base not strong enough to justify importance.

**Response**

 We expanded the literature base with **high-impact and mechanism-driven studies**.

**Revisions made**

 Added:

- Meta-analysis: Kaiser & Menkhoff (financial education effectiveness)
- Lusardi & Mitchell (financial literacy → economic outcomes)
- OECD reports (global benchmarking)
- Behavioral finance perspective (biases, heuristics)

**Strengthening strategy**

 We moved from:

 “financial literacy is important”

 To:

 “financial literacy operates through identifiable mechanisms affecting behavior, risk perception, and economic resilience”

**Impact**

- Stronger **theoretical justification**
- Eliminates “under-cited claim” weakness
- Aligns with **Scopus Q1 review expectations**

**Comment 3: Ambiguity of “financial literacy standards”**

**Reviewer concern:**

 Term “standards” unclear vs frameworks/competencies.

**Response**

 We clarified this concept explicitly and repositioned it analytically.

**Revisions made**

 Added definition:

 “Financial literacy standards in this study refer to structured competency benchmarks that define expected knowledge, skills, and behavioral capabilities in personal finance, as articulated in national (OJK) and international (OECD) frameworks.”

 Clarified distinction:

 TermMeaningStandardsBenchmark targets

 FrameworksConceptual structure

 CompetenciesMeasurable skills

**Impact**

- Removes conceptual ambiguity
- Improves **construct validity**
- Aligns with curriculum evaluation logic

**Comment 4: Weak theoretical grounding of financial literacy**

**Reviewer concern:**

 Needs stronger theoretical foundation.

**Response**

 We substantially reinforced the theoretical section.

**Revisions made**

 Integrated:

1. **OECD Financial Literacy Framework**
  - Knowledge
  - Behavior
  - Attitudes
2. **Behavioral Economics**
  - Bounded rationality
  - Financial decision biases
3. **Human Capital Theory**
  - Financial literacy as productive skill

**Inserted synthesis**

 Financial literacy is conceptualized as a multidimensional construct encompassing knowledge, behavior, and attitudes (OECD), shaped by cognitive limitations (behavioral economics), and functioning as a form of human capital influencing long-term economic outcomes.

**Impact**

- Upgrades from descriptive → **theoretically anchored**
- Strengthens **epistemic clarity**

**Comment 5: Methodological inconsistency (qualitative vs survey)**

**Reviewer concern:**

 Survey implies quantitative → inconsistency.

**Response**

 This is a critical point. We corrected positioning.

**Revisions made**

 We explicitly redefine design as:

**“qualitative-dominant mixed methods (QUAL + quan)”**

 Clarifications added:

- Survey = **supporting perception data**
- No statistical inference
- Used for **triangulation only**

**Inserted justification**

 The short survey serves as complementary evidence to support qualitative interpretation rather than as a basis for statistical generalization.

**Impact**

- Resolves **design inconsistency**
- Protects methodological coherence
- Prevents reviewer escalation

**Comment 6: Recommendations too general**

**Reviewer concern:**

 Need concrete examples / best practices.

**Response**

 We expanded this section significantly with applied cases.

**Revisions made**

 Added:

**Concrete examples**

1. **Financial Trading Rooms (FTR)**
2. **Simulation-based investment learning**
3. **Fintech-based budgeting tools**
4. **University–industry collaboration models**

**Example insertion**

 For instance, Financial Trading Rooms implemented in several universities allow students to engage with real-time market data, enabling experiential learning that significantly enhances financial decision-making skills.

**Impact**

- Moves from **generic → actionable**
- Improves **policy relevance**
- Enhances **practical contribution**

# Reviewer report: Reviewer response for version 1

**DOI:** 10.5256/f1000research.186083.r449277

**Published:** 2026-03-11

**Authors:** Nicholas Renaldo, Achmad Tavip Junaedi

**Affiliations:** 1 Institut Bisnis dan Teknologi Pelita Indonesia, Pekanbaru, Riau, Indonesia; 2 Institut Bisnis dan Teknologi Pelita Indonesia, Riau Province, Indonesia

1. The qualitative procedures are well described, but additional detail on sampling size and coding reliability would further improve replicability

 2. The study is qualitative and does not rely on statistical analysis

 3. The article is methodologically appropriate, clearly written, and well positioned for education, curriculum studies, and financial literacy journals. Revisions are mainly needed to sharpen theoretical framing, methodological transparency, and analytical depth, rather than to correct fundamental flaws.

 4. Results could be slightly more specific (e.g., explicitly naming “risk management” and “digital finance” as gaps).

 5. Explicitly state research questions (e.g., RQ1, RQ2) near the end of the introduction.

 6. Briefly anchor the analysis within one or two curriculum or learning theories to strengthen the conceptual foundation.

 7. Criteria for selecting Universitas Jambi and Universitas Pendidikan Indonesia should be clearer.

 8. The discussion largely restates results rather than deeply interpreting them.

 9. Recommendations are broad and sometimes generic.

 10. Reduce repetition with earlier sections.

Is the work clearly and accurately presented and does it cite the current literature?

Yes

If applicable, is the statistical analysis and its interpretation appropriate?

Not applicable

Are all the source data underlying the results available to ensure full reproducibility?

Yes

Is the study design appropriate and is the work technically sound?

Yes

Are the conclusions drawn adequately supported by the results?

Yes

Are sufficient details of methods and analysis provided to allow replication by others?

Partly

Reviewer Expertise:

Financial Literacy, Economics Education, Economics Curriculum

We confirm that we have read this submission and believe that we have an appropriate level of expertise to confirm that it is of an acceptable scientific standard, however we have significant reservations, as outlined above.

# Response

**Published:** 2026-03-11

**Authors:** Dwi Romadhon

**Affiliations:** FPEB, Universitas Pendidikan Indonesia, Bandung, West Java, Indonesia

We sincerely thank the reviewer for the constructive and insightful comments. The suggestions have helped improve the clarity, methodological transparency, and analytical depth of the manuscript. Revisions made in response to each comment are summarized below.

**1. Sampling size and coding reliability**

 Additional details have been added in the **Methodology section** to clarify the number of curriculum documents analyzed, interview participants, and student survey respondents. The coding procedure has also been clarified, including the use of a structured coding framework and expert discussion to ensure analytical consistency.

**2. Qualitative nature of the study**

 The manuscript now more explicitly states that the study employs **qualitative content analysis**, focusing on thematic interpretation rather than statistical testing.

**3. Strengthening theoretical framing and transparency**

 The conceptual framing and methodological explanations have been refined to improve clarity and analytical depth.

**4. More specific results**

 The **Results section** now explicitly highlights key gaps in the curriculum, particularly regarding **risk management, investment diversification, and digital financial tools**.

**5. Research questions**

 Clear research questions ( **RQ1–RQ3**) have been added at the end of the **Introduction** to guide the study.

**6. Theoretical anchoring**

 The analysis is now more clearly linked to **curriculum design principles and Bloom’s revised taxonomy** to strengthen the conceptual foundation.

**7. Institutional selection criteria**

 The **Methodology section** now explains the rationale for selecting **Universitas Jambi and Universitas Pendidikan Indonesia**, including program relevance and accessibility of curriculum documents for in-depth qualitative analysis.

**8. Deeper discussion**

 The **Discussion section** has been expanded to interpret the findings more analytically rather than simply restating the results.

**9. More specific recommendations**

 The recommendations have been refined to include concrete suggestions such as integrating **risk management, digital finance topics, experiential learning approaches, and industry collaboration**.

**10. Reducing repetition**

 The manuscript has been carefully edited to reduce repetitive statements and improve overall clarity.

 We appreciate the reviewer’s positive evaluation and believe these revisions have strengthened the manuscript.

# Reviewer report: Reviewer response for version 1

**DOI:** 10.5256/f1000research.186083.r436134

**Published:** 2026-01-06

**Authors:** Nadia Asandimitra Haryono

**Affiliations:** 1 Universitas Negeri Surabaya, Surabaya, East Java, Indonesia

The manuscript offers a timely and compelling examination of the extent to which economics education curricula in Indonesia genuinely equip students with the financial competencies required for contemporary economic life. The topic is highly relevant, particularly in light of the increasing global emphasis on financial literacy as a foundational life skill and the persistent critique that higher education institutions often privilege theoretical knowledge at the expense of practical, actionable financial capabilities. The study’s qualitative content‑analysis design is appropriate for its exploratory aims; however, the methodological narrative would benefit from greater precision and transparency. In particular, the manuscript should elaborate on the criteria used to select curriculum documents, the sampling strategy for educator interviews, and the analytic procedures employed to ensure rigor—such as coding reliability, triangulation, or reflexive validation.

 The findings are insightful and highlight a substantive gap between curricular content and the competencies required for effective financial decision‑making. The identification of underdeveloped areas—investment diversification, risk management, and digital financial tools—is particularly significant, given the rapid evolution of financial technologies and the growing complexity of personal finance. The students’ expressed desire for experiential learning modalities, including simulations, case‑based instruction, and industry collaboration, adds an important dimension to the analysis; however, the discussion would be strengthened by situating these insights within established theories of curriculum design, competency‑based education, and experiential learning. Moreover, the manuscript would benefit from a more explicit engagement with international benchmarks for financial literacy education, which could help contextualize Indonesia’s curricular gaps within broader global trends.

 The conclusion appropriately underscores the need for more applied, digitally oriented, and industry‑linked financial literacy training. Nonetheless, the implications section remains somewhat general and could be enhanced by offering more concrete, actionable recommendations for curriculum developers, policymakers, and higher education leaders. The acknowledgment of methodological limitations is appropriate, though the authors might further articulate how future research—particularly multi‑institutional, mixed‑methods, or longitudinal studies—could deepen the evidence base and enhance the generalizability of the findings.

 Overall, the manuscript addresses an important and underexplored dimension of economics education in Indonesia and demonstrates clear potential for scholarly contribution. Strengthening the theoretical framing, enhancing methodological clarity, and deepening the analytical discussion would significantly elevate the manuscript’s rigor and suitability for indexing in a Scopus‑indexed journal

Is the work clearly and accurately presented and does it cite the current literature?

Yes

If applicable, is the statistical analysis and its interpretation appropriate?

Yes

Are all the source data underlying the results available to ensure full reproducibility?

Yes

Is the study design appropriate and is the work technically sound?

Yes

Are the conclusions drawn adequately supported by the results?

Yes

Are sufficient details of methods and analysis provided to allow replication by others?

Yes

Reviewer Expertise:

behavioral finance

I confirm that I have read this submission and believe that I have an appropriate level of expertise to confirm that it is of an acceptable scientific standard.

# Response

**Published:** 2026-01-06

**Authors:** Dwi Romadhon

**Affiliations:** FPEB, Universitas Pendidikan Indonesia, Bandung, West Java, Indonesia

We thank the reviewer for the positive and constructive assessment of our manuscript. We appreciate the recognition of the study’s relevance, methodological soundness, and contribution to the field of economics and financial literacy education. The reviewer’s suggestions for further strengthening the theoretical framing and methodological clarity are well noted and will be carefully considered should revisions be required.

 Sincerely,

 Dwi Nanda Akhmad Romadhon

 (on behalf of the authors)
